# Dominance of *Bacillus cereus* and *Staphylococcus aureus* in Foodborne Outbreaks: A 19-Year Systematic Review of Surveillance Data in Yogyakarta, Indonesia

**DOI:** 10.3390/pathogens15090936

**Published:** 2026-09-04

**Authors:** Bayu Satria Wiratama, Arifah Alfi Maziyya, A’yun Hafisyah Wafi, Erica Yunita Trisnawati, Moch. Thoriq Assegaf Al-Ayubi, Shabrina Riskya Madjid, Sri Purwanti, Likke Prawidya Putri, Chih-Wei Pai

**Affiliations:** 1 Department of Biostatistics, Epidemiology and Population Health, Faculty of Medicine, Public Health and Nursing, Universitas Gadjah Mada, Yogyakarta 55281, Indonesia; arifahalfimaziyya@mail.ugm.ac.id (A.A.M.); ayunhafisyahwafi@mail.ugm.ac.id (A.H.W.); erica.yunita.t@mail.ugm.ac.id (E.Y.T.); thoriq.alayubi@gmail.com (M.T.A.A.-A.); shabrinariskya24m@gmail.com (S.R.M.); sripurwanti1995@mail.ugm.ac.id (S.P.); 2 Department of Health Policy and Management, Faculty of Medicine, Public Health and Nursing, Universitas Gadjah Mada, Yogyakarta 55281, Indonesia; likke.putri@ugm.ac.id; 3 Graduate Institute of Injury Prevention and Control, College of Public Health, Taipei Medical University, Taipei City 110, Taiwan; cpai@tmu.edu.tw

**Keywords:** foodborne outbreak, food handling, risk factors, Indonesia

## Abstract

Background/Objectives: Foodborne diseases account for the majority of infectious disease outbreaks in Yogyakarta, Indonesia, yet remain underexplored epidemiologically. This systematic review aimed to characterize the epidemiological patterns and risk factors of foodborne disease outbreaks in this region between 2006 and 2024. Methods: This systematic review searched electronic databases and grey literature published between 1 January 2006 and 31 December 2024. Eligible studies were reported in English or Indonesian and used descriptive or environmental observational designs. Methodological quality was assessed using a modified Joanna Briggs Institute (JBI) checklist. Searching, screening, and data coding were performed independently by the reviewers. The review followed PRISMA reporting guidelines and was registered with PROSPERO (CRD420251060794). Results: Of 841 studies screened, 99 met the inclusion criteria. The most frequently identified pathogens were *Bacillus cereus* (43.43%) and *Staphylococcus aureus* (34.34%), associated with 101 and 97 hospitalizations, respectively. Improper post-cooking storage (77.00%) was the leading contributing factor for *Bacillus cereus* outbreaks, whereas unsafe food processing practices (76.00%) predominated for *Staphylococcus aureus* outbreaks. Individual unregistered catering services accounted for the largest proportion of outbreaks. Conclusions: Public health strategies should prioritize strengthening food handlers’ capacity during food processing and post-cooking storage, particularly among individual informal catering services, to sharpen foodborne disease control efforts in Yogyakarta.

## 1. Introduction

Food poisoning, an acute illness resulting from consumption of contaminated food, is one clinical presentation of the broader category of foodborne disease (FBD). Each year, an estimated 600 million people worldwide experience FBDs, with 420,000 deaths [1]. Low- and middle-income countries bear a disproportionate share of this burden, with economic losses from healthcare spending and reduced productivity estimated at USD 110 billion annually [2]. Southeast Asia is the second most affected region after Africa, with around 150 million cases and 175,000 deaths each year [3]. Indonesia documented 353 food poisoning outbreaks (1100 people affected) in 2022, based on national surveillance data from the Indonesian National Agency of Drug and Food Control (BPOM) [4]. In the Special Region of Yogyakarta (Daerah Istimewa Yogyakarta), the official provincial administrative designation for this area under Indonesian law, food poisoning was also the most frequent cause of outbreaks, with 26 events recorded in 2023 [5].

An earlier assessment covering 2000–2015, preceding the period examined in this review, found that only 29.1% of reported outbreaks were fully investigated, indicating a longstanding limitation in surveillance and response capacity that motivates the present analysis. Several factors contributed to this gap, including delayed response, weak reporting systems, limited environmental investigation, and inconsistent outbreak protocols [6,7]. These constraints point to the need for stronger surveillance systems and more effective outbreak management.

The increasing number of outbreaks highlights the importance of improving food safety control. Systematic analysis of FBDs is essential for identifying risk factors, causative agents, and outbreak patterns, and supports early detection, raises public awareness, and informs policy decisions. However, many existing studies have methodological weaknesses: risk factors are often measured inconsistently, pathogen analysis is incomplete, and research coverage across regions is uneven, limiting representativeness. Most studies focus on common pathogens such as *Salmonella*, *Escherichia coli*, and *Norovirus* [8]. Research on environmental factors such as water quality and sanitation is also limited [6], and many studies do not examine food processing and storage conditions, including temperature control and distribution practices.

This systematic review therefore aims to provide a comprehensive understanding of foodborne illness patterns in Yogyakarta between 2006 and 2024 and to offer evidence-based recommendations for strengthening food safety measures. Specifically, it addresses the following review question: what are the epidemiological patterns—causative agents, outbreak characteristics, risk factors, and response timeliness—of foodborne disease outbreaks in the Special Region of Yogyakarta, Indonesia, between 2006 and 2024.

## 2. Materials and Methods

We conducted a systematic review in accordance with the Preferred Reporting Items for Systematic Reviews and Meta-Analyses (PRISMA) guidelines [9]. The review reports on outbreak magnitude (number of cases and attack rate), timeliness of response, incubation period, symptoms, case distribution, population characteristics, and causative agent and process. Eligible studies were full-text reports of food poisoning outbreaks published between 1 January 2006 and 31 December 2024. Eligible articles reported a food poisoning outbreak involving a population at risk residing in the Special Region of Yogyakarta, Indonesia. We excluded published articles describing the same population at risk as an included grey-literature source, as well as articles not published in English or Indonesian. The same eligibility criteria described above were applied uniformly to published articles and grey-literature sources. Eligible grey literature comprised outbreak surveillance records, health department reports, and records identified through citation searching. An outbreak was defined as an event involving two or more epidemiologically linked cases of acute illness following a shared food exposure, consistent with the case definition applied in the source surveillance systems. No minimum case threshold beyond this definition was applied. Outbreaks were eligible regardless of causative agent, including both microbiological pathogens and chemical hazards (e.g., histamine).

### 2.1. Search Strategy and Selection Criteria

We searched PubMed, Scopus, Web of Science Core Collection, ScienceDirect, and Portal Garuda for published articles, with a date restriction from 1 January 2006 to 31 December 2024. Searches were restricted to English and Indonesian languages (Portal Garuda). The full search strategies for each database are provided by request to corresponding author. In brief, we combined controlled vocabulary and free-text terms for “food poisoning” in both English and Indonesian (e.g., “Keracunan makanan,” “Keracunan pangan,” “Food poisoning”) using Boolean operators, with field restrictions appropriate to each database (e.g., [tiab] in PubMed; TITLE-ABS-KEY in Scopus). For English databases, we used (“food poisoning” OR foodborne OR “food-borne” OR “foodborne disease*” OR “foodborne illness*” OR “foodborne outbreak*” OR “food poisoning outbreak*” OR (gastroenteritis AND (food OR foodborne)) OR (diarrhea AND (food OR foodborne))). While for Indonesia database, we used (“keracunan makanan” OR “keracunan pangan” OR “wabah keracunan makanan” OR “keracunan makanan KLB” OR “penyakit bawaan makanan” OR (diare AND (makanan OR pangan)) OR (gastroenteritis AND (makanan OR pangan))). We also searched outbreak records from the Field Epidemiology Training Program residents’ report database, health department reports, and references of included studies as the grey literature. All authors screened titles and abstracts against the inclusion and exclusion criteria; selected publications then underwent full-text screening. Critical appraisal of each paper was conducted by all authors and subsequently discussed and reviewed by two authors (B.S.W. and C.W.P). The review protocol was registered with PROSPERO (registration number CRD420251060794. Each of the 99 included records was cross-checked against district, date of occurrence, and affected population to confirm that it represented a distinct outbreak event, consistent with the duplicate-population exclusion criterion applied during screening.

### 2.2. Data Extraction and Synthesis

Data were extracted on district and date of occurrence, food-handling setting, causative agent(s), case numbers and demographics, clinical outcomes, symptom profile, incubation period, contributing risk factors, and response timeliness. Data were synthesized descriptively; no inferential hypothesis testing was performed. Data extraction was performed by A.A.M., A.H.W., E.Y.T., M.T.A.A.-A., S.R.M., and S.P.

### 2.3. Quality Assessment and Data Analysis

All authors conducted a critical appraisal for quality assessment, covering methodological quality and risk of bias. We used a modification of the Joanna Briggs Institute (JBI) checklist for prevalence studies, previously applied in a similar study in India [10], to assess the credibility and consistency of included sources (Table S1). The checklist comprised seven items. Studies meeting five or more of the seven criteria were classified as low risk of bias; studies meeting three or four criteria were classified as moderate risk of bias; and studies meeting two or fewer criteria were classified as high risk of bias. Disagreements in risk-of-bias assessment were resolved by the first author (B.S.W.) through discussion. Descriptive quantitative results are presented as percentages and frequencies, calculated using Percentages were calculated using the number of outbreaks or cases with available information on the relevant variable as the denominator, rather than the full sample of 99 outbreaks or 8563 cases, unless otherwise stated. The specific denominator is reported alongside each percentage in Section 3. Descriptive quantitative results are presented as percentages and frequencies, calculated using STATA 17 software.

Generative artificial intelligence (Perplexity AI ver 16/04/2026) was used solely to improve the grammar, clarity, and language quality of the manuscript text; it was not used for study design, data collection, analysis, or interpretation. The authors reviewed and edited all AI-assisted output and take full responsibility for the content of the manuscript.

### 2.4. Ethics Approval

This systematic review received approval from the Institutional Review Board of the Faculty of Medicine, Public Health and Nursing, Universitas Gadjah Mada (KE/FK/0880/EC/2025). No competing financial interests or personal relationships influenced the work reported in this paper. All authors were responsible for the decision to submit the manuscript. Ethical approval was obtained because this review accessed non-public outbreak surveillance records held by district health offices, in addition to published sources. These records contained aggregated case information without personal identifiers. The review protocol was registered with PROSPERO (CRD420251060794); no amendments were made to the registered protocol.

## 3. Results

### 3.1. Study Selection Process

**Figure 1:**
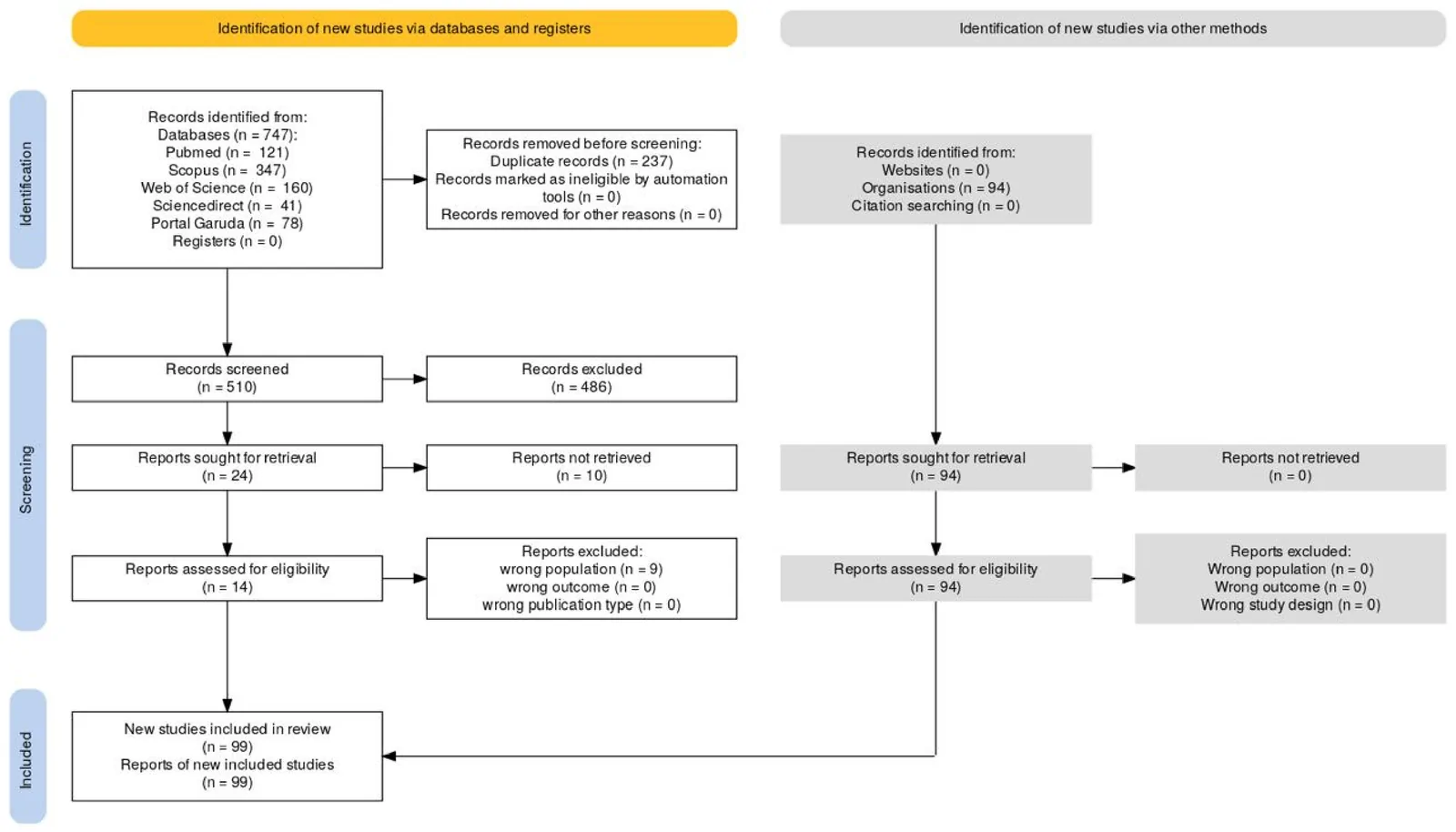
PRISMA Flow Chart.

Figure 1 shows the PRISMA flow diagram for this review. The systematic literature search identified 747 records, of which 510 unique records underwent title and abstract screening. Of these, 486 records were excluded, 10 reports could not be accessed, and 9 reports were excluded for wrong population (non-Yogyakarta cases) or other eligibility issues, leaving 5 included reports from database searches. An additional 94 studies meeting full eligibility criteria were identified through other methods, primarily organizational disease surveillance records, health department reports, and citation searching. In total, 99 studies documenting 99 distinct food poisoning outbreaks in the Special Region of Yogyakarta between 2006 and 2024 were included in the final analysis.

### 3.2. Quality Assessment of Included Studies

The large majority of included studies (95.2%) were assessed as having a low risk of bias; one study was classified as moderate risk of bias and four as high risk of bias. Studies with low risk of bias reported a lower median number of cases than those with high risk of bias (53 vs. 77 cases). This finding suggests a generally low risk of bias across included sources, although this assessment should be interpreted with caution given that the appraisal tool was a modified checklist applied predominantly to outbreak reports and grey-literature sources rather than peer-reviewed epidemiological studies.

### 3.3. Geographic and Temporal Distribution

Table 1 reveals important district-level epidemiological heterogeneity: Sleman and Bantul districts showed a high proportion of paediatric cases (90.1% and 94.5% of cases <18 years, respectively) and comparatively short mean incubation periods (5.2 and 7.5 h, respectively); this descriptive combination of characteristics was not a pre-specified classification.

**Figure 2:**
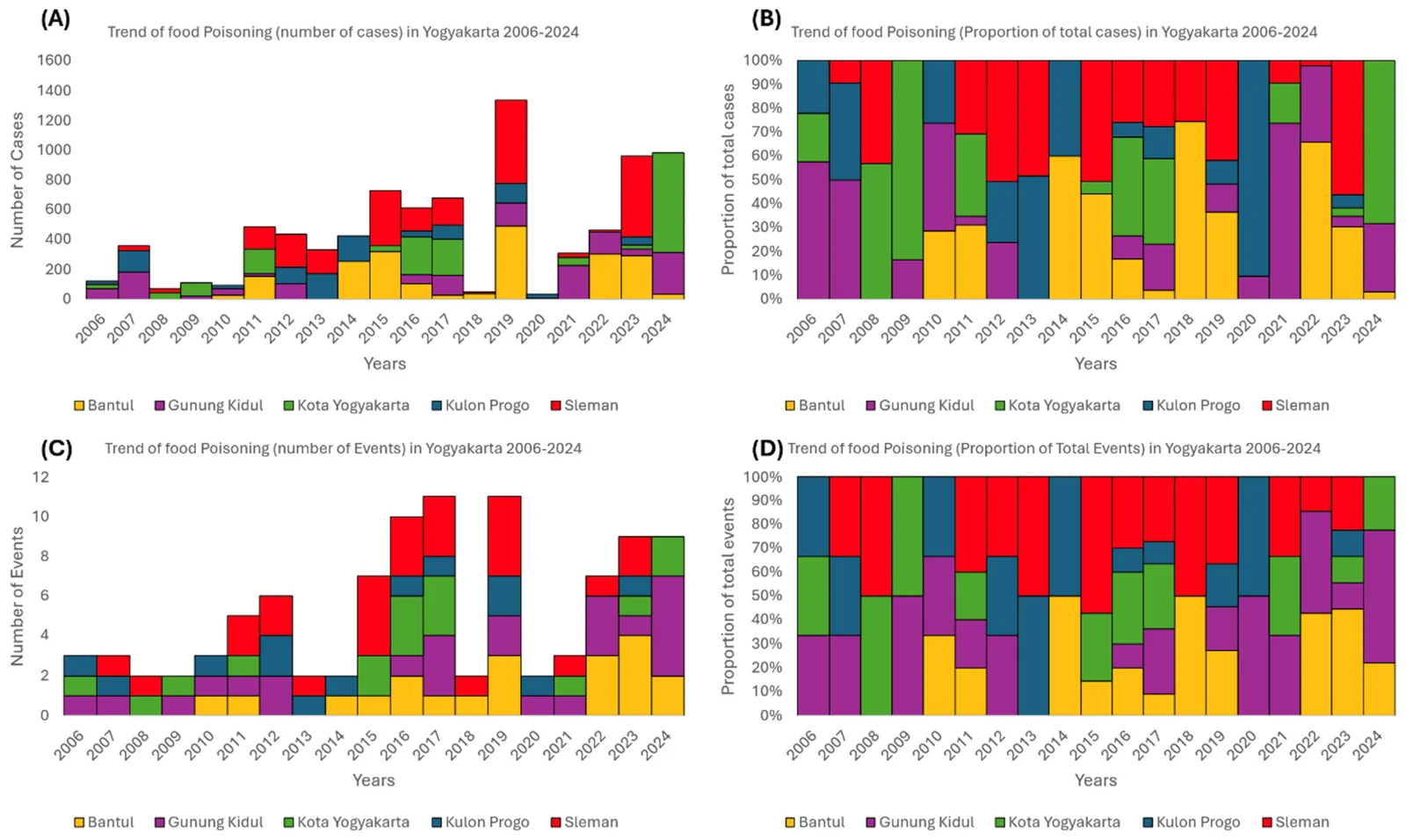
Trend of food poisoning in Yogyakarta 2006–2024: (**A**) trend of number of cases; (**B**) trend of total cases proportion; (**C**) trend of number of events; and (**D**) trend of total events proportion.

Figure 2 illustrates the rise in outbreak events and cases over time, with a notable increase during 2022–2024, suggesting a post-pandemic rebound following the relaxation of COVID-19 mobility restrictions. During 2015–2017, outbreak events increased without a corresponding rise in total illness cases; this pattern could reflect a true epidemiological change (e.g., smaller average outbreak size) or may instead be related to differences in surveillance sensitivity, reporting completeness, or outbreak investigation practices during this period, which cannot be distinguished with the available data. Sleman, Bantul, and Gunung Kidul consistently accounted for the largest proportion of reported outbreaks. The proportional trend in case numbers also shows a geographical shift: during 2006–2009, cases were concentrated in Yogyakarta City and Gunung Kidul, whereas in later years the proportion of reported cases shifted toward Bantul and Sleman, based on the descriptive proportional trends shown in Figure 2B,D; population denominators were not available to express this as a rate.

#### Causative Agents

**Figure 3:**
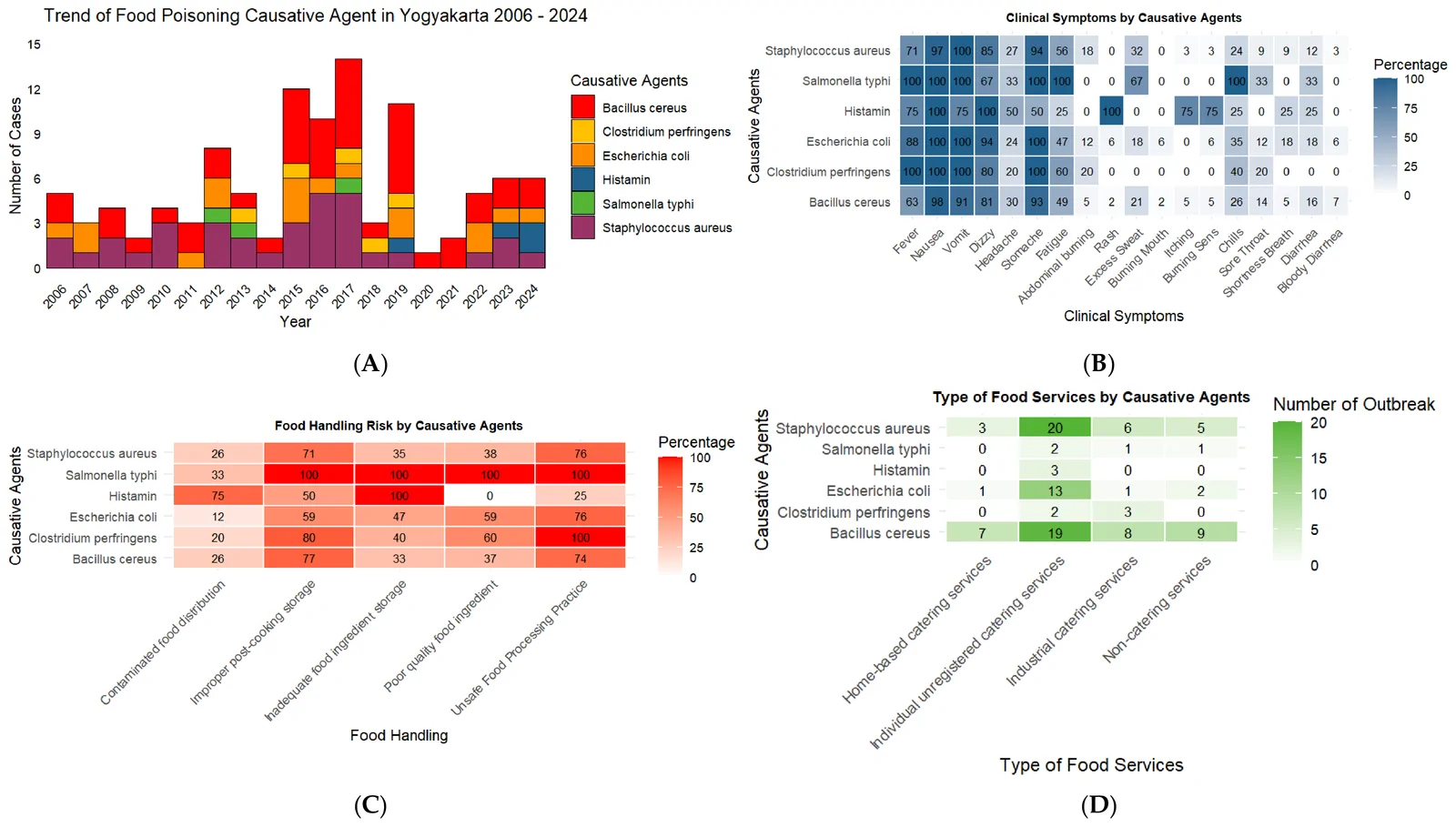
Characteristics of Food Poisoning Based on Causative Agent: (**A**) Trend of Food Poisoning Causative Agent in Yogyakarta 2006–2024; (**B**) Clinical Symptoms by Causative Agents; (**C**) Food Handling Risk by Causative Agents; (**D**) Type of Food Services by Causative Agents.

Causative-agent categories are not mutually exclusive, as multiple agents were identified in some outbreaks (21 outbreaks; 21.21%) and no agent was identified in others (20 outbreaks; 20.20%); percentages are therefore calculated on a denominator of 99 outbreaks and do not sum to 100%. Trends in causative agents over time were predominantly driven by *Bacillus cereus* and *Staphylococcus aureus*. Histamine-related outbreaks began to emerge after 2018, whereas outbreaks caused by *Salmonella typhi* were seldom reported after 2017 (Figure 3). Overall, *Bacillus cereus* accounted for 43 outbreaks (43.43%), followed by *Staphylococcus aureus* with 34 outbreaks (34.34%). Gram-negative pathogens (*Escherichia coli*, 17 outbreaks, 17.17%; *Salmonella typhi*, 3 outbreaks, 3.03%) and the Gram-positive, spore-forming anaerobe *Clostridium perfringens* (5 outbreaks, 5.05%) were less prevalent than the two dominant agents. As a chemical hazard rather than a biological pathogen, histamine intoxication was documented in 4 outbreaks (4.04%), all associated with consumption of scombroid fish or other high-histamine foods. No causative agent was identified in 20 outbreaks (20.2%), and multiple agents were identified in 21 outbreaks (21.2%). The identification of multiple etiological agents in some outbreaks suggests either polyetiological contamination from multiple food sources or concurrent infections within the affected populations.

### 3.4. Attack Rates, Outcomes, and Response Timeliness

The 99 outbreaks collectively affected 8563 individuals (Table 1). The mean outbreak size was 86.5 cases (SD 114.9), with a median of 60 cases (IQR 28–110), ranging from 3 to 636 cases per outbreak. Outcome data were available for 3586 of 8563 cases (41.9%): outpatient treatment in 39.20% of all cases (3357/8563), hospitalisation in 3.76% of all cases (322/8563), and one death (case fatality rate 0.012% of all cases). Outcome status was not recorded for the remaining 58.10% of cases (4977/8563). According to the causative agents presented in Table 2, the three agents contributing the highest absolute number of hospital admissions were *Bacillus cereus* (101/3148 cases; 3.21%), *Staphylococcus aureus* (97/2929 cases; 3.31%), and histamine (71/618 cases; 11.49%); notably, histamine showed the highest hospitalisation rate among all agents despite the smallest absolute case count. Regarding outbreak location, and defining response timeliness as the interval in hours between outbreak occurrence and initiation of investigation, Bantul demonstrated the fastest initial response, with an average interval of 9.6 h between occurrence and the initiation of investigation. Bantul, Yogyakarta City, and Gunung Kidul consistently maintained an initial response time of less than 24 h from reported onset. In contrast, Kulon Progo exhibited a markedly longer response time, averaging 70.2 h.

### 3.5. Host Characteristics

Age information was available for 2213 of 8563 cases (25.80%); age data were missing for the remaining 6350 cases (74.20%). Among cases with recorded age information, the age distribution showed a paediatric predominance; given the substantial missing data, this distribution may not be representative of all outbreak-associated cases: children <18 years, 1328 cases (60.0%); adults 18–59 years, 744 cases (33.60%); and elderly ≥60 years, 141 cases (6.40%). Among 7856 cases with recorded sex data, females predominated, accounting for 57.30% (n = 4499) of outbreak-associated cases. The median incubation period across all 99 outbreaks, irrespective of causative agent, was 7.0 h (IQR 2.0–12.0 h), with substantial variation (range 0–79.5 h) reflecting the diversity of etiological agents involved; this figure numerically coincides with, but is distinct from, the *Bacillus cereus*-specific mean incubation period of 7 h reported in Table 2.

### 3.6. Clinical Presentation

**Figure 4:**
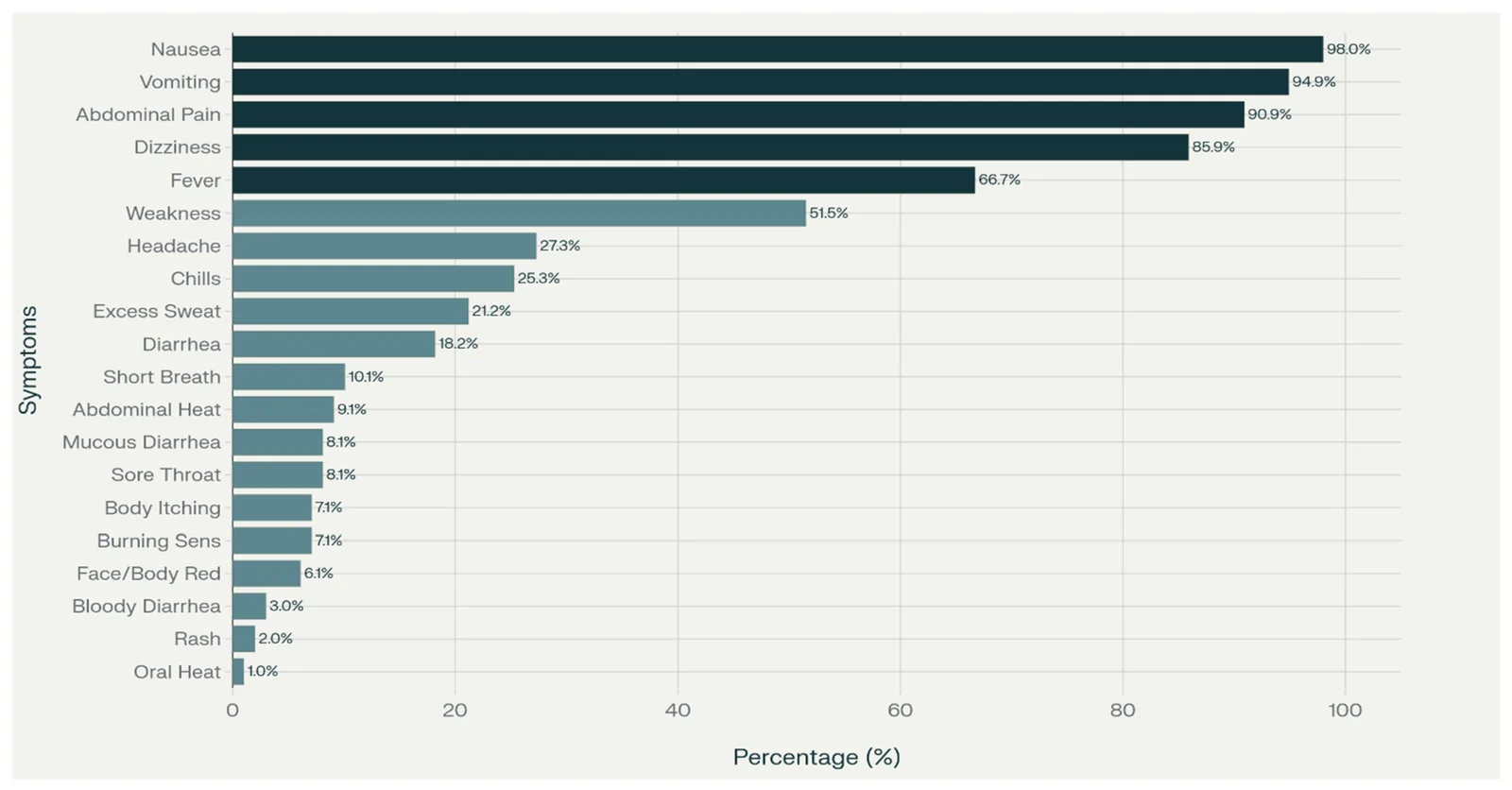
Clinical Symptoms in Food Poisoning in Yogyakarta (n = 99), Ranked by Frequency.

Gastrointestinal manifestations predominated across all reported outbreaks. The most frequently observed symptoms (Figure 4) were nausea (reported in 97.9% of the 99 outbreaks, i.e., 97 outbreaks; a total of 4592 cases across those outbreaks with case-level symptom data available), vomiting (94.8% of outbreaks; 2331 cases), and abdominal pain (90.6% of outbreaks; 4434 cases). Percentages refer to the proportion of outbreaks in which each symptom was documented as present; case counts are reported only for outbreaks with case-level symptom data and are not available for all outbreaks in which the symptom was noted qualitatively. Respiratory and dermatological symptoms were comparatively uncommon, occurring in fewer than 10% of outbreaks. In outbreaks caused by *Bacillus cereus*, nausea (98%) and abdominal symptoms (93%) were commonly reported. Vomiting (100%) and nausea (97%) were more frequently observed in *Staphylococcus aureus* outbreaks. Histamine-related outbreaks were primarily characterized by rash, whereas cases associated with *Salmonella typhi* commonly presented with chills (Figure 4).

### 3.7. Incubation Period by Etiological Agent

Mean incubation period (calculated across outbreaks attributed to each causative agent, as reported in Table 2) varied substantially across causative agents, reflecting differences in underlying pathogenic mechanisms. Histamine intoxication had the shortest incubation period (approximately 1 h). *Staphylococcus aureus* outbreaks demonstrated a mean incubation period of 5 h, and *Bacillus cereus* of 7 h. Longer incubation intervals were observed for *Clostridium perfringens* (13 h) and *Escherichia coli* (13.5 h). *Salmonella typhi* had the longest incubation period (16 h).

### 3.8. Environmental and Food-Handling Risk Factors

Environmental factors were involved in the majority of outbreaks, with food processing issues the most frequent contributing factor (72 outbreaks; 72.72%), including inadequate temperature control during cooking, insufficient heating, and cross-contamination during preparation (Table 3). By causative agent, *Bacillus cereus* outbreaks (n = 43) were most often attributed to improper post-cooking storage (33/43 outbreaks; 77%), particularly failure to maintain appropriate temperatures, inadequate refrigeration, and prolonged holding of prepared foods at ambient temperature, whereas *Staphylococcus aureus* outbreaks (n = 34) were more frequently linked to unsafe food processing practices (26/34 outbreaks; 76%). Histamine outbreaks (n = 4) were attributed to inadequate food ingredient storage (4/4; 100%) and contaminated food distribution (3/4; 75%).

### 3.9. Food-Handling Settings

Analysis of outbreak settings further highlighted the vulnerability of domestic and small-scale food-handling environments. More than half of all outbreaks occurred in individual unregistered catering services (59 outbreaks; 55.66%), followed by industrial catering services (19 outbreaks; 17.92%), non-catering services such as school canteens or street snack vendors (17 outbreaks; 16.04%), and home-based catering services (5 outbreaks; 5.1%) (Table 4). The predominance of individual unregistered catering services is discussed further in Section 4.

## 4. Discussion

Among the subset of cases with recorded clinical outcome data (41.9% of all cases), this review found a low proportion of hospitalisation and mortality, alongside a female predominance, with *Bacillus cereus* as the dominant causative agent. Prior research has documented that females are more likely to be involved in outbreaks linked to vegetable consumption, an environment favorable to *Bacillus* contamination [11]; this may reflect food preferences associated with gender [12]. Children under 18 years were the most vulnerable population, likely reflecting lower stomach acid, an immature immune system, smaller body mass, and less control over their own meals [13]. The low hospitalisation rate and mortality observed among cases with recorded outcomes may reflect a comparatively low disease severity, although incomplete outcome reporting (58.1% of cases with no recorded outcome) cannot be excluded as a contributing explanation and limits confidence in this interpretation; timely response has previously been shown to reduce the risk of foodborne disease transmission [14]. Bantul had the fastest response time among the districts studied, whereas Kulon Progo had the longest; longer response times can be influenced by geographic conditions, shortages of surveillance officers, and limited resources [15].

The most frequently observed symptoms—nausea, vomiting, and abdominal pain—likely reflect the causative agents involved. Our two leading causative agents, *Bacillus cereus* and *Staphylococcus aureus*, produce toxins with emetic activity and enteropathogenic effects that cause nausea, vomiting, diarrhoea, and abdominal pain [16]. *Bacillus cereus* has been reported to show a global prevalence of approximately 36–41% during 1990–2020, with the heaviest burden in Asian and other developing regions [17]. In Indonesia, *B. cereus* similarly ranks as the third most common biological pathogen, with prevalence reaching 26.7% between 2000 and 2024 [4,5,6,7]. In contrast, *S. aureus* shows a comparatively lower global prevalence, ranging from 18% to 35% during 2012–2022 depending on food source [18]; a similar pattern has been documented in Indonesia, with reported prevalence in the range of 18–30% over the same period [4,5,6,7]. These trends collectively suggest that both pathogens continue to play a substantial role in foodborne illness across diverse geographic and temporal contexts. These comparator prevalence estimates are derived from food-sample or clinical-isolate testing studies and use different denominators from the outbreak-level proportions reported in the present review; they are presented here as contextual reference points rather than directly comparable measures.

The predominance of upper-gastrointestinal symptoms (nausea and vomiting) observed in this review’s leading causative agents, *Bacillus cereus* and *Staphylococcus aureus*, is consistent with the emetic-toxin mechanism shared by both organisms, in which preformed heat-stable toxin acts directly on the gastric mucosa and vagal afferents to induce rapid emesis rather than the invasive or enterotoxigenic mechanisms that produce predominantly lower-gastrointestinal (diarrhoeal) symptoms in *Escherichia coli* and *Salmonella typhi* outbreaks. A plausible contextual contributor to the predominance of emetic-toxin pathogens in this setting is ambient-temperature holding of cooked foods in Indonesian food-service settings, including rice dishes, which can create conditions favourable for toxin-producing spore-formers such as *Bacillus cereus* [19,20].

A plausible contextual contributor to the emetic-toxin pathogen predominance in this setting is the common practice of holding pre-cooked rice and coconut-based dishes at ambient temperature in Indonesian catering, conditions that favour toxin-producing spore-formers.

Our finding of a 17.17% prevalence for *Escherichia coli* is lower than national estimates reported for Indonesia over 2000–2024 (45–74.9%) [4,6,7]. Internationally, *E. coli* is frequently identified as a major contributor to foodborne illness: it accounted for 48.82% of foodborne pathogen cases in China between 2011 and 2022, ranked among the top four pathogens by case count in the United States and the European Union between 1998 and 2017, and was the leading cause of foodborne disease in Republic of Korea between 2010 and 2018 [1,21,22]. Some regional variation has nonetheless been observed; Turkey reported a lower prevalence of 12.27% in 2024, and Japan showed a declining trend between 2000 and 2018 [1,23]. These discrepancies may reflect differences in surveillance systems, diagnostic capacity, food supply chains, or regulatory enforcement across countries [1]. As with the comparisons above, these international *E. coli* prevalence estimates are drawn from food-sample or clinical-isolate testing studies with different denominators from the outbreak-level proportion reported here and should be read as contextual reference points rather than directly comparable measures.

As a chemical hazard rather than a biological pathogen, histamine was identified as a notable contributor, accounting for 4.04% of foodborne disease outbreaks in this review (4 of 99 outbreaks; Table 2). This proportion is comparable to Indonesia’s reported histamine prevalence of 6.7% during 2000–2024 [4,7]. Although histamine-related incidents are generally less frequent than bacterial outbreaks, their persistence suggests that chemical hazards remain an important part of the food safety landscape, particularly in fish and fishery products where histamine formation is most likely to occur [24]. Histamine poisoning in this review had the shortest incubation period, consistent with investigations in Seoul, Republic of Korea, and in France, which reported a median incubation period of 60 min [25,26]. Several histamine poisoning cases indeed tend to show very rapid onset [8]. The Seoul and Reunion Island studies were selected as comparators because they are, to our knowledge, the only prior peer-reviewed reports providing a quantified incubation period for scombroid/histamine poisoning, not because of any shared population characteristics with Yogyakarta.

In this review, the incubation period for *Bacillus cereus* was 7 h, in line with the reported range of 6–24 h attributable to toxin production following spore germination [8]. For *E. coli*, the incubation period was 13.5 h, although *Shiga* toxin–producing strains may take up to 4 days [27]. This discrepancy should be interpreted cautiously rather than assumed to reflect the typical incubation period of this pathogen in this setting (see Limitation paragraph). *Clostridium perfringens* can cause incubation periods of up to 24 h, whereas symptoms in this review appeared after 12 h [28]; both processes involve bacterial growth and subsequent toxin release or mucosal invasion. The incubation period for *Staphylococcus aureus* was 5 h in this review, compared with 9.5 h reported elsewhere, consistent with rapid absorption of heat-stable enterotoxins [29]. The longest incubation period, for *Salmonella*, is reported elsewhere in the range of 6 to 72 h, reflecting its capacity for systemic invasion [6]. Similarly, the incubation period we observed for *Salmonella typhi* (16 h) is markedly shorter than internationally reported ranges (6–72 h), and this discrepancy should be interpreted cautiously rather than assumed to reflect the typical incubation period of this pathogen in this setting (see Limitation paragraph).

These variations highlight the importance of assessing the incubation period during epidemiological investigation to help identify likely etiological agents in a timely manner. Pathogen identification remains necessary, since different organisms—and even different strains—can produce different incubation periods and clinical features. The etiologic conclusion is strongest when epidemiological evidence, laboratory results, environmental findings, and pathogen confirmation align.

Food processing issues were the most frequently reported contributing factor to food poisoning outbreaks in Yogyakarta. This finding aligns with a study conducted in Malaysia, which reported a food poisoning outbreak involving 21 cases in which improper cooking and reheating practices were the leading cause (57.1%); insufficient cooking and reheating can promote bacterial growth [30]. Inadequate cooking temperatures and insufficient cooking time can allow pathogenic bacteria to proliferate; these organisms can generally be controlled by maintaining temperatures below 4 °C or eliminated at temperatures above 70 °C [31,32]. Evidence from previous studies, together with the present analysis, suggests that inadequate food temperature control is a consistent and persistent causative factor in outbreaks [33,34].

The predominance of individual unregistered catering services among outbreak settings in this review reflects structural challenges commonly reported in informal food-service sectors, including limited access to reliable refrigeration, inadequate sanitation infrastructure, lower levels of food safety knowledge and training among food handlers, and the absence of systematic regulatory oversight compared with registered catering establishments.

Improper food storage can further contribute to food poisoning outbreaks by enabling cross-contamination and bacterial proliferation. A comparable pattern, although in a different food-handling context, was reported in an outbreak in Northern Thailand, where storing cooked and raw food in the same container facilitated bacterial growth, with *S. aureus* identified as the causative agent [35]. Similarly, a *Clostridium perfringens* outbreak in England was linked to improper storage, in which cooked and raw vegetables were placed in the same container, compounded by inadequate sanitation; separating raw and cooked food can help prevent foodborne illness [36]. These international examples are presented as illustrative parallels rather than direct confirmation of the mechanisms underlying the Yogyakarta outbreaks, as differences in food-handling systems and surveillance context between settings limit direct generalisability.

The category of process failure includes process irregularities, improper cooking, inadequate temperature control during preparation, and improper packaging. Mislabelling can also contribute to food poisoning by allowing errors in expiration dating [37]. A listeriosis outbreak linked to cantaloupe melons illustrates the consequences of improper distribution: trucks used to dispose of melon waste at cattle ranches were parked next to packaging facilities, allowing contamination to spread, particularly in areas where standing water was present [38].

The strengths of this review relate primarily to the methodological quality of the included evidence and the comprehensive scope of the analytic synthesis. The large majority of included studies (95.2%) were assessed as having a low risk of bias using the modified JBI checklist; this should be interpreted with appropriate caution given that the checklist was adapted rather than used in its original validated form, and was applied predominantly to outbreak reports and grey-literature sources. The synthesis also provides insight into both biological causative agents and environmental risk factors, particularly deficiencies in food processing practice, linking etiological agents with contextual determinants of outbreaks. In addition, the review identifies vulnerable populations—notably children under 18 years, who accounted for 60.0% of cases with available age information (1328 of 2213 cases with recorded age; 25.8% of all 8563 cases had age recorded)—and maps geographic shifts in outbreak burden across districts in Yogyakarta over time.

This review has several limitations. First, data extraction relied primarily on a single data recording system; reports from other institutions (e.g., BPOM) were not available. Second, the absence of a fully standardized recording and reporting system across institutions may have contributed to inconsistencies and variability in data availability, potentially affecting completeness and comparability. Third, outcome data (outpatient treatment, hospitalization, or death) were available for only 41.9% of cases; the remaining 58.1% had no outcome category recorded in either the source reports or the underlying extraction data. Fourth, incubation period estimates for *E. coli* and *Salmonella typhi* diverged substantially from previously published ranges for these pathogens, for reasons that could not be resolved with the data available and that may reflect pathotype differences, ascertainment timing, or original misclassification. Fifth, only five of the 99 included records were identified through the formal database searches, with the substantial majority (94 records) identified through organisational surveillance records, health department reports, and citation searching; this heavy reliance on non-database sources may limit the reproducibility of the search process by independent researchers and should be considered when interpreting the completeness of this review. These limitations should be weighed against the strengths of the review. Sixth, the specific food vehicle implicated in each outbreak was not consistently recorded as a discrete, codeable variable across the 99 sources, precluding a systematic food-item-by-pathogen analysis; treatment modality and duration of recovery were similarly under-reported and could not be reliably synthesised across sources. These variables are recommended targets for future prospective surveillance. The review’s strengths include its 19-year scope, the high overall methodological quality of included studies, and its status as the first synthesis specific to this region over this period.

## 5. Conclusions

This review shows that *Bacillus cereus* and *Staphylococcus aureus* were the most frequent causative agents of foodborne disease outbreaks in Yogyakarta between 2006 and 2024. A rising trend in both outbreaks and cases over the past five years coincided with frequently reported weaknesses in food processing and storage practices. We also found variation in outbreak response time across regions of Yogyakarta. Strengthening hygiene and sanitation measures, improving the capacity of food handlers—particularly among individual informal catering services—and standardizing response-time thresholds remain essential for reducing the risk of foodborne disease and preventing future outbreaks in Yogyakarta. Response-time thresholds should be informed by the substantial district-level variation observed in this review (average initial response ranging from 9.6 h in Bantul to 70.2 h in Kulon Progo); an illustrative target of response within 24 h of outbreak onset is consistent with the performance already achieved by Bantul, Yogyakarta City, and Gunung Kidul in this review.

## Figures and Tables

**Table 1:** Outbreak distribution by district, case numbers, sex, age, incubation period, and response timeliness.

District	Outbreaks	Total Cases	Male (%)	Female (%)	<18 y (%)	18–59 y (%)	≥60 y (%)	Incubation (h)	Response (h) *
Sleman	26	2456	867 (36.4%)	1512 (63.6%)	418 (90.1%)	13 (2.8%)	33 (7.1%)	3.0 (0.2–20)	34.2
Gunung Kidul	24	1480	534 (36.6%)	927 (63.4%)	159 (24.1%)	438 (66.3%)	64 (9.7%)	10.0 (0–34)	21.0
Bantul	20	2021	755 (42.7%)	1015 (57.3%)	600 (94.5%)	24 (3.8%)	11 (1.7%)	8.0 (0–20)	9.6
Yogyakarta City	16	1611	840 (51.9%)	780 (48.1%)	95 (55.6%)	45 (26.3%)	31 (18.1%)	6.5 (0.5–16)	18.0
Kulon Progo	13	995	501 (51.8%)	466 (48.2%)	56 (19.9%)	224 (79.4%)	2 (0.7%)	8.0 (0–79.5)	70.2
Total	99	8563	3497 (42.7%)	4700 (57.3%)	1328 (60.0%)	744 (33.6%)	141 (6.4%)	7.0 (0–79.5)	28.1

* Average interval, in hours, between outbreak occurrence and initiation of investigation.

**Table 2:** Causative agent distribution among foodborne disease outbreaks.

Causative Agent	Outbreaks	%	Total Cases	Hospital Admission	Ambulatory Care	Mean Incubation (h)
Biological pathogens						
*Bacillus cereus*	43	43.43	3148	101	1186	7
*Staphylococcus aureus*	34	34.34	2929	97	1226	5
*Escherichia coli*	17	17.17	1657	54	744	13.5
*Clostridium perfringens*	5	5.05	740	6	114	13
*Salmonella typhi*	3	3.03	318	4	119	16
Chemical hazard						
Histamine	4	4.04	618	71	114	1

Causative-agent categories are not mutually exclusive, as multiple agents were identified in some outbreaks (21 outbreaks; 21.21%) and no agent was identified in others (20 outbreaks; 20.20%); percentages are calculated on a denominator of 99 outbreaks and do not sum to 100%.

**Table 3:** Environmental and food-handling risk factors during outbreaks (not mutually exclusive; an outbreak may have more than one contributing factor).

Risk Factor	Outbreaks	%
Unsafe food processing practice	72	72.72
Improper post-cooking storage	59	59.59
Poor quality food ingredients	34	34.34
Inadequate food ingredient storage	34	34.34
Contaminated food distribution	23	23.23

**Table 4:** Distribution of food-handling setting.

Food Handling Type	Outbreaks	%
Individual unregistered catering services	59	55.66
Industrial catering services	19	17.92
Non-catering services (school canteen/street snack vendors)	17	16.04
Home-based catering services	11	10.38

## Data Availability

Data will be available upon request to the corresponding author.

## Supplementary Materials

The following supporting information can be downloaded at https://www.mdpi.com/article/10.3390/pathogens15090936/s1. Table S1: Critical Appraisal Tool. Table S2: PRISMA 2020 checklist.

## Abbreviations

The following abbreviations are used in this manuscript:

FBD	Foodborne disease
PRISMA	Preferred Reporting Items for Systematic Reviews and Meta-Analyses
JBI	Joanna Briggs Institute
IRB	Institutional Review Board
PROSPERO	International Prospective Register of Systematic Reviews
IQR	Interquartile range
SD	Standard deviation
BPOM	Badan Pengawas Obat dan Makanan (Indonesian National Agency of Drug and Food Control)
CDC	Centers for Disease Control and Prevention
