# Supplementary material for: Dominance of Bacillus cereus and Staphylococcus aureus in Foodborne Outbreaks: A 19-Year Systematic Review of Surveillance Data in Yogyakarta, Indonesia
Source: Pathogens. 2026 Sep 4;15(9):936. doi: 10.3390/pathogens15090936 (PMC13610027; doi:10.3390/pathogens15090936)
Supplement: Supplementary file 1 [file pathogens-15-00936-s001.zip › Table S1.pdf]

Table S1. Critical Appraisal Tool

Modified JBI Critical Appraisal Checklist	Criteria items and scores (0/0.5/1) by relevance
1. Was there a mention of an outbreak report*	Mention of an outbreak/epidemic in the title/ abstract; Yes=1/No=0
2. Was there a case definition for inclusion of participants *	Mention of a case definition based on standard available case definitions or clinical symptoms in case of unknown conditions; Yes=1, No=0
3. Was the condition identified and measured in a standard, reliable way for all participants	Based on mention of a lab diagnosis/ clinical criteria/ epidemiologically linked for the confirmed/suspected cases; Yes=1, No=0
4. Were the study subjects described in detail?	Mention of participants as case patients, If only numerators mentioned the =0.5 and if mentioned by numerator and denominator =1(case attack rate), if no mention = 0
5. Was the study setting and the outbreak time period mentioned in detail	A detailed mention of the study setting of the outbreak and the time of onset of the outbreak, last case.<br>When Study setting/ Time of the outbreak is mentioned =0.5<br>When study setting and time period of the outbreak mentioned (even if only date of onset)=1<br>When neither study setting or time period of outbreak mentioned =0
6. Was there an appropriate analysis?*	Analysis in the report can be of descriptive nature or analytic based on the study design. The methods section should be detailed enough for reviewers to identify the analytic technique used and how specific variables were measured Mention of percentages, case fatality, Odds Ratio or Relative Risk, Population Attributable Risk (PAR) should be clearly reported, confidence intervals If relevant any of the above is mentioned Yes=1/No=0
7. Were the inferences made based on the observation and hypothesis *	Mention of relevant public health inferences (in term of prevention, diagnosis, treatment, environment, social) from the results Yes=1/No=0

Quality	Score
HIGH	All the 4 criteria covered and mark 7
MEDIUM	One of the 4 criteria covered
LOW	If None of the 4 criteria covered
